# Temporal and Within-Sporophyte Variations in Triphenyltin Chloride (TPTCL) and Its Degradation Products in Cultivated *Undaria pinnatifida*

**DOI:** 10.3390/plants13060767

**Published:** 2024-03-08

**Authors:** Xingyue Ren, Yuanyuan Zhang, Xu Gao, Qingli Gong, Jingyu Li

**Affiliations:** Key Laboratory of Mariculture, Ministry of Education, Fisheries College, Ocean University of China, Qingdao 266003, China; renxingyue1998@163.com (X.R.); yyzhang@ouc.edu.cn (Y.Z.); qingli@vip.sina.com (Q.G.)

**Keywords:** triphenyltin chloride, accumulation, degradation, *Undaria pinnatifida*

## Abstract

*Undaria pinnatifida* can effectively deal with organotin pollution through its excellent accumulation and degradation capabilities found under laboratory conditions. However, nothing is known regarding its accumulation, degradation performance, and related impact factors in the wild farming area. In this study, we monitored triphenyltin chloride (TPTCL) contents and degradation products in different algal parts (blades, stipes, sporophylls, and holdfasts) of cultivated *U. pinnatifida* from December 2018 to May 2019. Our results showed that sporophytes had an accumulation and degradation capacity for TPTCL. The TPTCL contents and degradation products varied with the algal growth stages and algal parts. TPTCL accumulated in the blades at the growth stage and the blades, stipes, sporophylls, and holdfasts at the mature stage. The TPTCL content among algal parts was blades (74.92 ± 2.52 μg kg^−1^) > holdfasts (62.59 ± 1.42 μg kg^−1^) > sporophylls (47.24 ± 1.41 μg kg^−1^) > stipes (35.53 ± 0.55 μg kg^−1^). The primary degradation product DPTCL accumulated only in the blades at any stage, with a concentration of 69.30 ± 3.89 μg kg^−1^. The secondary degradation product MPTCL accumulated in the blades at the growth stage and in the blades, stipe, and sporophyll at the mature stage. The MPTCL content among algal parts was blades (52.80 ± 3.48 μg kg^−1^) > sporophylls (31.08 ± 1.53 μg kg^−1^) > stipes (20.44 ± 0.85 μg kg^−1^). The accumulation pattern of TPTCL and its degradation products seems closely related to nutrient allocation in *U. pinnatifida*. These results provide the basis for applying cultivated *U. pinnatifida* in the bioremediation of organotin pollution and the food safety evaluation of edible algae.

## 1. Introduction

Triphenyltin chloride (TPTCL) is an artificial synthetic compound with the composition of tin atoms and phenyl [1,2], and is widely used in wood preservatives, antifouling coatings, and agricultural pesticides [3,4]. Industrial and domestic wastewater are two principal pathways of bringing TPTCL into the marine ecosystem and, therefore, antifouling, causing significant damage to marine organisms due to its powerful biocidal characteristics [5,6,7]. For marine animals, TPTCL has mainly led to biomorphological changes in oysters [8], imposex in gastropods [9,10,11], and neurobehavioral toxicity in fish [12]. For marine microalgae, TPTCL forces the alteration of physiological processes through membrane lipid interactions destroying internal photosynthetically active lamellae or inhibiting nitrate reductase activity [13,14]. Nevertheless, there have been a limited number of investigations regarding the physiological responses of marine macroalgae to TPTCL stress.

*Undaria pinnatifida* (Laminariales; Phaeophyceae) is a forest-forming brown macroalga indigenous to the Northwestern Pacific area [15,16,17] and has recently established a presence across temperate rocky coasts worldwide [18,19,20]. This species plays an important role in the maintenance of coastal water environments by removing excessive nutrients [21,22] and alleviating chemical pollution [23]. Additionally, *U. pinnatifida* has been maricultured on a large scale in East Asia [24,25,26] because of its great commercial value in foodstuffs [27,28], pharmaceuticals, and agriculture [29,30,31,32]. In recent years, coastal chemical pollutants have constituted a major environmental threat to seaweed farming [33]. Our previous study showed that high TPTCL concentrations negatively influenced the survival, growth, and enzyme activities of cultivated sporophytes of *U. pinnatifida*, promoting a decline in yield and quality [34]. Additionally, *U. pinnatifida* sporophytes exhibited excellent TPTCL accumulation and degradation capabilities under controlled laboratory conditions. Therefore, it was considered an appropriate candidate for bioremediation [35]. Nevertheless, TPTCL’s accumulation and degradation patterns in the species as well as the relative impact factors have not been investigated under field cultivation conditions.

In the present study, we investigated changes in TPTCL and its degradation products in cultivated *U. pinnatifida* at different life stages and parts within sporophytes. These study results are expected to provide important information for assessing the seawater purification potential of this high-value species in China. These results are expected to help evaluate the food safety of this commercial species.

## 2. Results

### 2.1. Seasonal Variation in Water Temperature and Nutrient Concentration

**Figure 1:**
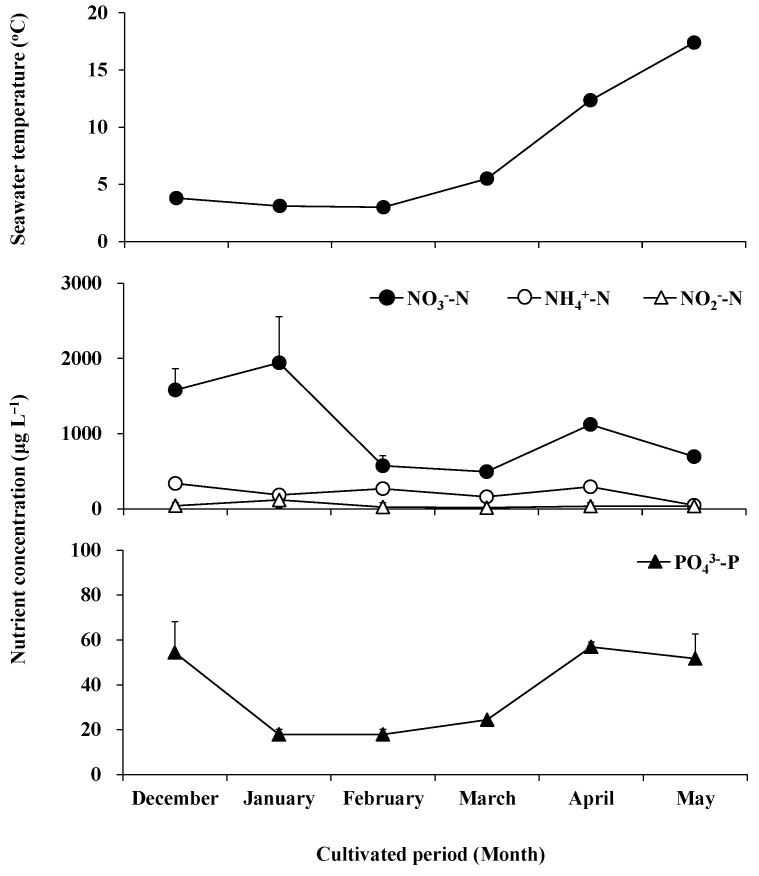
The water temperature and concentrations of nitrate (solid circle), ammonium (clear circle), nitrite (clear triangle), and orthophosphate (solid triangle) in seawater during field cultivation from December 2018 to May 2019.

**Figure 2:**
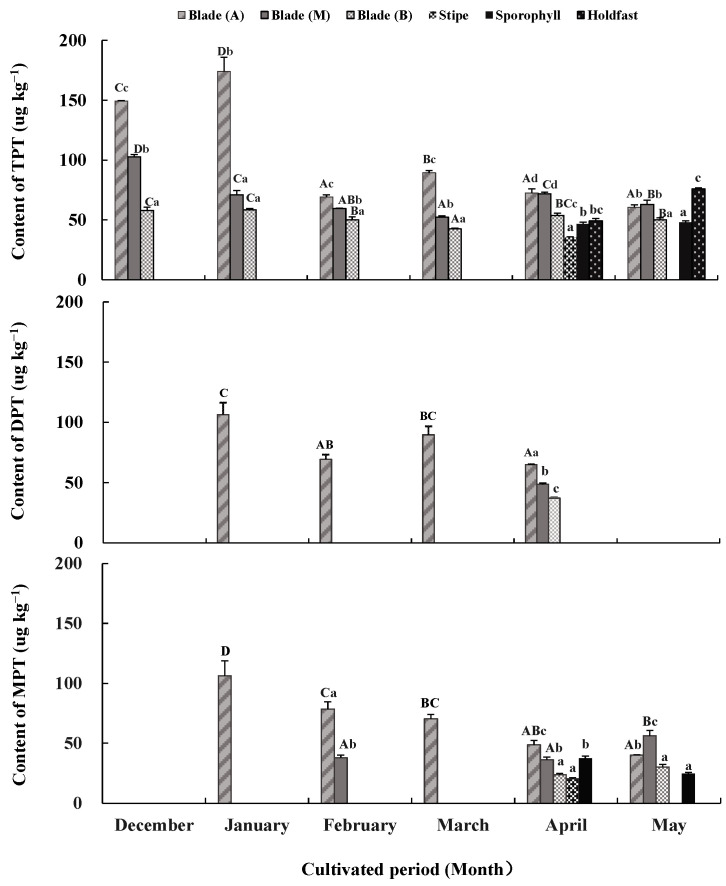
Triphenyltin chloride (TPTCL) content and its degradation products diphenyltin dichloride (DPTCL) and monophenyltin trichloride (MPTCL) in *Undaria pinnatifida* tissues during field cultivation. Different capital and lowercase letters represent significant differences among algal parts and cultivated periods, respectively, at *p* < 0.05.

The water temperature varied slightly from 3 °C to 5.5 °C from December to March, and rose rapidly in April and May, reaching 12.4 °C and 17.4 °C, respectively (Figure 1). The fluctuation of NO_3_^−^-N concentration was greater than that of NO_2_^−^-N, NH_4_^+^-N, and PO_4_^3−^-P (Figure 2). The NO_3_^-^-N concentration increased from December to January, reaching an annual maximum of 1942.51 μg L^−1^ in January, decreased from 494.63 μg L^−1^ to 573.44 μg L^−1^ in February and March, recovered to 1121.87 μg L^−1^ in April, and decreased again to 695.67 μg L^−1^ in May. The NO_2_^−^-N concentration was maintained between 19.49 μg L^−1^ and 44.56 μg L^−1^ in months other than January, where its concentration reached a maximum of 120.70 μg L^−1^. The NH_4_^+^-N concentration maintained a range of 185.98 μg L^−1^–337.96 μg L^−1^ from December to April and decreased to a minimum of 47.47 μg L^−1^ in May. The PO_4_^3-^-P concentration was 54.41 μg L^−1^ in December, decreased from 18.01 μg L^−1^ to 24.49 μg L^−1^ between January and March, and returned to 51.69 μg L^−1^–56.87 μg L^−1^ in April and May.

### 2.2. Seasonal Morphological Characteristics of U. pinnatifida

Table 1 shows the morphological characteristics (length, width, and fresh weight) of blades, stipes, and sporophylls in cultivated *U. pinnatifida*. Significant growth was shown in the morphological features of blades and stipes from December 2018 to February 2019 (*p* < 0.05). The length and width values increased and reached a peak of 138.33 ± 27.06 cm and 22.54 ± 5.99 cm at the blades and 38.96 ± 10.78 cm and 1.86 ± 0.29 cm at the stipes in February. Afterward, these values decreased in March, slightly rebounded in April, and in May reached another peak of 101.88 ± 20.97 cm and 36.89 ± 9.39 cm at the blades and 247.36 ± 133.77 cm and 36.61 ± 8.37 cm at the stipes. Changes in fresh weight were similar to those of length and width. The fresh weight of the blades and stipes reached a maximum of 247.36 ± 133.77 g and 93.73 ± 32.10 g in May after experiencing fluctuations in December and April. Sporophylls appeared in February, and their length, width, and fresh weight gradually increased and reached a maximum of 28.02 ± 11.70 cm, 6.49 ± 1.62 cm, and 105.95 ± 72.5 g, respectively, in May.

### 2.3. TPTCL and Its Degradation Products in Seawater and in Sporophytic Tissues from Different Parts

TPTCL and its degradation products DPTCL and MPTCL were not detected in the seawater samples but were detected in sporophytic tissues (Figure 2). TPTCL, DPTCL, and MPTCL concentrations varied across algal tissues and the cultivated periods (*p <* 0.05).

From December to March, TPTCL was only detected in blade tissue. Its content was generally higher in apical tissue (69.22 μg kg^−1^–174.08 μg kg^−1^), followed by middle tissue (52.29 μg kg^−1^–102.70 μg kg^−1^), and basal tissue (42.52 μg kg^−1^–58.46 μg kg^−1^) (*p <* 0.05). In April and May, TPTCL was detected in almost all algal tissues, except for stipes in May. The order of TPTCL content from large to small in each algae tissue was apical and middle blade tissues (71.74 μg kg^−1^–72.52 μg kg^−1^) > basal blade tissue, holdfasts and sporophylls (46.57 μg kg^−1^–53.71 μg kg^−1^) > stipes (35.53 μg kg^−1^) in April, and holdfasts (75.84 μg kg^−1^) > apical and middle blade tissues (60.72 μg kg^−1^–63.01 μg kg^−1^) > basal blade tissue and sporophylls (47.90 μg kg^−1^–50.23 μg kg^−1^) in May (*p <* 0.05). Compared to the values from December to March, the gap among the tissue of different algal parts was reduced in April and May. There was also a significant difference in the TPTCL content between the cultivated periods. The TPTCL content in the apical blade tissue fluctuated greatly, reaching its maximum in January (174.08 μg kg^−1^), followed by December (149.29 μg kg^−1^) and March (89.49 μg kg^−1^), and remaining low in other months (60.72 μg kg^−1^–72.52 μg kg^−1^) (*p <* 0.05). The TPTCL content in the middle and basal blade tissues decreased from December to March and rebounded slightly in April and May (*p <* 0.05); however, its fluctuation was not as significant as that of the apical blade tissue. TPTCL content in the stipe tissue still existed in April but disappeared in May, whereas TPTCL content in the holdfast increased from April to May.

DPTCL, the primary degradation product of TPTCL, was detected only in apical blade tissue from January to March and in minor amounts in all blade tissues in April. DPTCL content in January (106.28 μg kg^−1^) was the highest, followed by that in March (89.74 μg kg^−1^), with the lowest in February and April (37.03 μg kg^−1^–69.35 μg kg^−1^) (*p <* 0.05). The average DPTCL content during the cultivated period was 69.30 ± 3.89 μg kg^−1^ in blade tissue.

MPTCL, the secondary degradation product of TPTCL, only appeared in apical or middle blade tissues from January to March. It also appeared in nearly all test tissues in April and May. The TPTCL content in the apical and middle blade tissues was always higher than in other tissues (*p <* 0.05). The maximum MPTCL content was in January in the apical blade tissue (106.23 μg kg^−1^), and this value decreased gradually until May (40 μg kg^−1^) (*p <* 0.05). The MPTCL content in the middle blade tissue was kept low in February and April but increased significantly in May (56.19 μg kg^−1^) (*p <* 0.05). The MPTCL content in other tissues fluctuated in the range of 20.44 μg kg^−1^–37.51 μg kg^−1^. The average MPTCL content was 52.80 ± 3.48 μg kg^−1^ in blade tissue, followed by 31.08 ± 1.53 μg kg^−1^ in sporophylls and 20.44 ± 0.85 μg kg^−1^ in stipes.

## 3. Discussion

### 3.1. Accumulation of TPTCL by Cultivated U. pinnatifida

TPTCL content was detected in most sporophyte parts of *U. pinnatifida* throughout the cultivated period, although it was not detected in the seawater collected in the cultivation area. TPTCL content accumulated only in the blades from December to March and in most algal parts from April to May. The average TPTCL content throughout the cultivated period was 74.92 ± 2.52 μg kg^−1^ in the blades, followed by 62.59 ± 1.42 μg kg^−1^ in holdfasts, 47.24 ± 1.41 μg kg^−1^ in sporophylls, and 35.53 ± 0.55 μg kg^−1^ in stipes. These results suggested that *U. pinnatifida* has a remarkable bioaccumulation capability for TPTCL. The bioconcentration factor (BCF) is an important indicator for describing the magnitude of bioaccumulation of organic compounds in organisms [36]. Unfortunately, it failed to obtain accurate BCFs of *U. pinnatifida* from TPTCL because TPTCL content in seawater could not be detected. To compare the BCFs between different algal parts and species, we estimated the BCFs of *U. pinnatifida* using the detection limit we established for TPTCL in seawater samples. The BCFs of TPTCL between different parts of *U. pinnatifida* were approximately >386.02 in blades (>528.36 in apical blades, >360.83 in middle blades, and >268.88 in basal blades), >322.44 in holdfasts, >243.35 in sporophylls and >183.07 in stipes. In this study, the BCFs of TPTCL by *U. pinnatifida* were much lower than those previously reported in microalgae and macroalgae. The BCFs of TPT by the freshwater green microalga *Tetradesmus obliquus* (formerly alga *Scenedesmus obliquus*) were 1.14 × 10^5^. The BCFs of TBT by *T. obliquus* and the mixed microalgae *Dunaliella salina* and *D. viridis* were >3.32 × 10^5^ and >3.48 × 10^5^, respectively, after a 7-day exposure to TPT [37]. The BCFs of TBT by marine microalgae *Chaetoceros neogracilis* (formerly *Chaetoceros gracilis*) (Mediophyceae), *Platymonas* sp. (Chlorophyta), and *Phaeodactylum tricornutum* (Bacillariophyceae) were 4.53 × 10^5^, 5.9 × 10^4^, and 9.1 × 10^4^, respectively, when exposed to TBT for 72 h [38]. The TBT and TPT concentrations in the phytoplankton of Otsuchi Bay, Japan, were 240 μg kg^−1^–980 μg kg^−1^ and 20 μg kg^−1^–670 μg kg^−1^ dry weight; the BCFs were 3.0 × 10^3^–1.32 × 10^5^ and 2.2 × 10^3^–7.44 × 10^4^, respectively [39]. There were few reports on TPT and TBT accumulations by macroalgae except for our previous study. TPTCL and TBTCL content in the young sporophytes of *U. pinnatifida* reached a maximum of 1659.87 μg kg^−1^ and 2973.84 μg kg^−1^ after 3 days of exposure to 5.0 μg L^−1^ of TPTCL and TBTCL; the BCFs were >8.55 × 10^3^ and >2.97 × 10^4^, respectively. TPTCL and TBTCL concentrations in the matured sporophytes of *U. pinnatifida* were 1704.17 μg kg^−1^ and 1650.63 μg kg^−1^; the BCFs were >8.78 × 10^3^ and >8.25 × 10^3^, respectively [35]. BCFs can be affected by pollutant properties (type, concentration, structure, and existence form), biological characteristics of algae (species, size, sex, organ, growth and development stage), and environmental conditions (temperature, salinity, water hardness, dissolved oxygen concentration, and light conditions) [40]. In water, suspended particles and sediments have strong adsorption of all kinds of trace contaminants, affecting their existence, form, bioavailability, and bioaccumulation [1]. Microalgae, a type of suspended particle, have a higher ratio of surface area to volume and more advantages in surface adsorption and organotin uptake than macroalgae [38]; therefore, they have more BCFs than macroalgae. *U. pinnatifida* cultured in the laboratory exhibits higher BCFs of TPTCL than *U. pinnatifida* cultivated in the field because of the high initial concentration of TPTCL and stable culture conditions in the laboratory, including temperature, light and water velocity, and nutrient supply [35]. Although the BCFs of cultivated *U. pinnatifida* were not as high as microalgae, they are still expected to accumulate a large amount of TPTCL from seawater in the cultivation process because of its huge production (an output of 225,600 tons of fresh weight in 2021) [41] and long cultivation period (from October to May every year). These results suggest that cultivated *U. pinnatifida* has great potential as a bioremediation tool for removing organotin compounds from natural seawater, especially in waters with severe organotin pollution.

### 3.2. Degradation of TPTCL by Cultivated U. pinnatifida

In February, there was a sharp reduction (about 30~55%) in TPTCL content in the blades, especially in the apical part of the blades. Afterward, the TPTCL content in the algal parts remained at relatively low levels until May. Subsequently, DPTCL, the primary degradation product of TPTCL, appeared in the blades from January to April, and its content gradually decreased. MPTCL, the secondary degradation product of TPTCL, appeared in the blades and other parts from January to May, but its content gradually reduced. DPTCL and MPTCL appeared one month later than TPTCL, whereas DPTCL disappeared one month earlier than MPTCL. Furthermore, the appearance of both DPTCL and MPTCL was accompanied by a decrease in TPTCL, indicating that DPTCL and MPTCL appeared with the gradual degradation of TPTCL by *U. pinnatifida*. DPTCL and MPTCL appeared simultaneously and reached their maximum concentrations in January. However, only TPTCL and MPTCL were retained in the algal tissues in May. These results revealed that TPTCL degradation by *U. pinnatifida* was more efficient at the growth stage than at the mature stage.

Previous studies on organotin degradation by algae mainly focused on TBT rather than TPT. Fifty percent of TBT was degraded to DBT, MBT, and inorganic tin by a green alga *Ankistrodesmus falcatus* when exposed to a certain TBT concentration for 4 weeks [42]. *Chaetoceros neogracilis* degraded 5% TBT into DBT and MBT after exposure to 0.4 μg L^−1^ of TBT for 72 h [38]. *Chlorella vulgaris* degraded 27% and 41% of TBT into DBT and MBT, whereas *Chlorella* sp. only degraded 26% of TBT into DBT when exposed to 100 μg L^−1^ and 30 μg L^−1^ of TBT for 14 days [43]. The four microalgae, *Chlorella miniata*, *C. sorokiniana*, *Tetradesmus dimorphus* (formerly *Scenedesmus dimorphus*), and *Comasiella arcuata var. platydisca* (formerly *Scenedesmus platydiscus*) degraded TBT into DBT and MBT inside the cells. TBT-specific uptake and degradation by *Chlorella* was higher than by *Tetradesmus*/*Comasiella*, likely due to larger cell sizes and biomass [44]. Both the microalgae *Leptocylindrus danicus* (Mediophyceae) and *Amphidinium carterae* (Dinophyceae) degraded TBT to the less toxic DBT and MBT. *A. carterae* transformed DBT into MBT more rapidly than *L. danicus* [45]. Our previous study was the first to report TPTCL and TBTCL degradation by the macroalgae *U. pinnatifida* [35]. *U. pinnatifida* exposed to TPTCL and TBTCL for 12 days absorbed 100% of TPTCL and 98% of TBTCL in the culture medium and degraded them into DPTCL and DBTCL and, subsequently, DPTCL and DBTCL into MPTCL and MBTCL. The TPTCL and TBTCL concentrations and their degradation products were MPTCL > TPTCL > DPTCL for sporophytes at any stage, TBTCL > DBTCL for the young stage, and TBTCL > DBTCL > MBTCL for the mature stage. *U. pinnatifida*’s ability to degrade TPTCL was higher than that of TBTCL, and its degradation capacity at the mature stage was stronger than at the growth stage. These results revealed that algae’s capacity to degrade TPTCL and TBTCL varies with algal species, growth and development stages, cell compositions, enzyme activities, and organotin forms and concentrations.

Regarding *U. pinnatifida*’s degradation mechanism for TPTCL and TBTCL, whether cultivated in the field or in the laboratory, no degradation products were detected in seawater, assuming that degradation occurs in the algae cells. It was acknowledged that the degradation of organotin compounds by micro-organisms mainly occurred in the cell but not on the surface of the cell wall [45,46]. TPTCL is transferred across the cell membrane through active transport and interactions with membrane lipids and proteins due to its hydrophobicity and metabolism [47], causing intracellular accumulation and biodegradation. TPTCL dephenylation may also be related to the cellular metabolism of ions, carbohydrates, and organic acids [46]. Metabolite analysis confirmed that TPTCL was degraded through the cleavage of Sn-C bonds producing diphenyltin, monophenyltin, and tin, respectively [48]. The Sn-C bonds could be effectively cleaved by cytochrome P450, which is the enzyme responsible for TPTCL degradation [49]. Unfortunately, most of TPTCL’s degradation mechanisms were obtained from micro-organisms but not from marine algae. More studies should identify TPTCL’s degradation mechanisms in marine algae.

TPT and TBT are well known for their strong toxicity and significant impact on marine environments and organisms [50]. *U. pinnatifida*’s strong degradation capacity for TPTCL and TBTCL guarantees its application in the bioremediation of organotin pollution. In addition, *U. pinnatifida* is also a commercial species for food or food additives; therefore, its food safety has also attracted more attention. In this study, the average TPTCL content in *U. pinnatifida* cultivated in Jiaozhou Bay from December to the following May varied between 57.99 μg kg^−1^ and 103.35 μg kg^−1^ in blades, 0 μg kg^−1^ and 35.53 μg kg^−1^ in stipes, and 47.95 μg kg^−1^ and 61.87 μg kg^−1^ in sporophylls. According to the acceptable daily intake (ADI, 0.5 μg kg^−1^ body weight per day) of TPTCL regulated by the FAO and WHO [51,52], a person with 60 kg of body weight should consume 0.29 kg–0.52 kg of fresh blades, or 0.84 kg–no limit of stipes, or 0.48 kg–0.63 kg of sporophylls daily. The blades appear to be more risky than other algal parts, especially in December and January. Food safety evaluations of cultivated *U. pinnatifida* should consider multiple factors, such as the cultivation area, organotin concentration, algal growth stage, algal parts and so on.

### 3.3. Temporal and Intra-Sporophyte Variations of TPTCL and Its Degradation Products in Cultivated U. pinnatifida

TPTCL accumulation and its degradation products in cultivated *U. pinnatifida* varied with cultivation periods and algal parts. TPTCL accumulation was more active from December to January, and DPTCL and MPTCL accumulations were more active from January to March. The presence of TPTCL and its degradation products was concentrated only in blades from December to March, and in minor amounts in blades, sporophylls, holdfasts, and stipes from April to May. TPTCL’s accumulation pattern is consistent with the allocation and storage pattern of nutrients, such as carbon and nitrogen, in *U. pinnatifida*. Previous reports demonstrated that TPTCL transport and stepwise transformation are metabolically mediated activities in aquatic animals [53,54]. Other results certified that TPTCL degradation by microbes was related to the cellular metabolism of ions, carbohydrates, organic acids, and enzymes [46,49]. These results suggest that TPTCL’s transportation and transformation may depend on intracellular nutrients, such as carbon and nitrogen, which help explain TPTCL’s accumulation pattern and nutrient allocation pattern. The cultivated *U. pinnatifida* grew rapidly from December to February. Nutrients produced through photosynthesis and nutrient uptake were mainly allocated and stored in the blades and midribs (recorded as part of the stipes) to expand productive areas and improve production. With the appearance of sporophylls in February, nutrients were mainly allocated and stored in the basal part of the blades (with meristem) and sporophylls in April and May to prepare for growth in width, thickness, and reproduction. The midway harvest of larger individuals led to a sharp reduction in March and a slow increase in April of morphological parameters and induced developmental stagnation of sporophylls and regrowth of the blades in width and thickness (Table 1). Similar growth and nutrient allocation patterns were reported in *U. pinnatifida* cultivated in Miyagi, Japan, after thallus excision. Thallus excision caused compensatory growth, first in the blades and then in sporophylls [55]. TPTCL and its degradation products in *U. pinnatifida* had similar temporal and intra-sporophyte variations to nutrients, suggesting that TPTCL can indicate nutrient allocation patterns.

In addition, some algal parts of *U. pinnatifida* such as holdfasts and sporophylls with no or little productivity had high TPTCL contents and degradation products, implying a long-distance transport of nutrients in these algae. To meet the meristem’s nitrogen and carbon demands, the long-distance transport of nitrogen and carbon from mature to basal blades has been reported in the brown macroalgae *Saccharina japonica* (formerly *Laminaria japonica*), *Laminaria digitata*, *Laminaria hyperborea,* and *Saccharina latissima* (formerly *Laminaria saccharina*) [56,57,58,59]. Another report noted that some brown algae in Laminariaceae, Lessoniaceae, and Alariaceae could translocate assimilates from the non-growing part toward the intercalary growing region, the stipes, and even holdfasts, to support new tissue formation and reproductive organs [60]. The sieve elements of the medulla were considered the transport route [59]. This long-distance transport mechanism may explain intra-sporophyte variations in TPTCL and its degradation products in cultivated *U. pinnatifida*.

## 4. Materials and Methods

### 4.1. Sample Collection and Treatment

**Figure 3:**
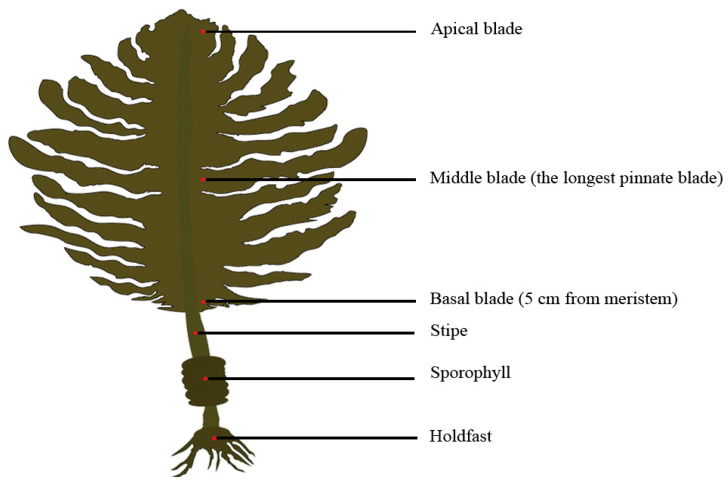
The algal parts of *Undaria pinnatifida* from which sporophytic tissues were excised for the detection of TPTCL and its degradation products.

From December 2018 to May 2019, *U. pinnatifida* sporophytes (*n* = 70) were randomly collected every month from cultivated populations in Jiaozhou Bay (36° 06′ N, 120° 18′ E), Qingdao, China. At the same time, 500 mL of seawater samples were collected every month from the culture area’s surface (at a depth of 0.5 m). The seawater temperature was measured in situ with a thermometer. The algal and seawater samples were transported immediately to the laboratory in cooling boxes. Forty-five healthy sporophytes were chosen and fully rinsed with sterilized filtered seawater to remove detritus and epiphytes. There were three replicates in this investigation, with each replicate comprising 15 sporophytes. The length and width of the blades, stipes and sporophylls were measured for each sporophyte. The fresh weight of each part was measured after artificial segmentation. For each sporophyte, 5 g of fresh tissue was excised from holdfasts, sporophylls, stipes, basal blades (5 cm from meristem), middle blades (the longest pinnate blade), and apical blades, respectively (Figure 3). These algal parts and seawater samples were promptly placed in a freezer and stored at a temperature of −80 °C for subsequent experiments. In addition, concentrations of NO_3_^−^-N, NO_2_^−^-N, NH_4_^+^-N, and PO_4_^3−^-P in seawater samples were determined according to the Specifications for Oceanographic Survey (GB/T 12763.4-2007) (Ministry of Natural Resources of China 2007) [61].

### 4.2. Determination of TPTCL and Its Degradation Products in Seawater and Sporophytic Tissues from Different Parts

Eight organotin chloride standards, namely triphenyltin (TPT, 95%), diphenyltin (DPT, 96%), monophenyltin (MPT, 98%), tributyltin (TBT, 99%), dibutyltin (DBT, 95%), monobutyltin (MBT, 98%), dimethyltin (DMT, 98%), and tetrabutyltin (TeBT, 95%), with internal standard tripropyltin (TPrT, 99.3%) and the derivative agent sodium tetraethylborate (NaBEt4, 98%), were purchased from Beijing Bellingway Technology Co., Ltd. (Beijing, China). The concentrations of these reagents were 1000 mg (Sn) L^−1^ for organotin standards and 36.25 μg L^−1^ for TPrT, respectively. Tropolone (98%) was purchased from Tokyo Chemical Industry Co., Ltd. (Tokyo, Japan), and other reagents used in analysis were purchased from Sinopharm Chemical Reagent Co., Ltd. (Shanghai, China).

To determine the concentrations of TPTCL and its degradation products in seawater, 20 mL of seawater and 100 μL of TPrT standard solution were poured into a 50 mL glass tube and maintained for 30 min under dark conditions. To extract TPTCL and its deg-radation products, 10 mL of acetic acid–sodium acetate buffer solution, 300 μL of NaBEt4 (2%), and 2 mL of n-hexane were added to the tube and mixed intensively using an oscillator for 5 min. The upper extract was collected. We added and further mixed 2 mL of tropolone-n-hexane (0.005%) for 2 min and the upper extract was recovered. All these extracts were mixed and evaporated into 1 mL.

Before extracting TPTCL and its degradation from sporophytic tissues, lyophilized seaweed samples were ground into a powder and filtered through a 40-mesh sieve (380 μm). After fully mixing, 0.3 g dry powder and 100 μL TPrT standard solution were placed in flasks and maintained overnight under dark conditions. To extract TPTCL and its degradation products, 5 mL of NaCl solution (20%) and 15 mL of HCL-ethyl acetate (0.3 mol L^−1^) were added to flasks and mixed intensively using an ultrasonic treatment for 15 min at 32 kHz. We added and stirred 20 mL of tropolone-n-hexane (0.01%) for 40 min. The mixture was centrifuged at 6000 rpm for 10 min and the upper extract was collected. Subsequently, 15 mL of n-hexane was added to the remaining deposit and stirred for 20 min for the second extraction. We added 20 mL of acetic acid–sodium acetate buffer solution, 300 μL of NaBEt4 (2%), and 1.5 mL of n-hexane to the extracts and stirred for 30 min. These extracts were mixed and evaporated into 1 mL.

After passing through the 0.22 μm organic system filter membrane, the organic phases were prepared for loading onto a gas spectrum. A gas chromatograph (SCION 456-GC, Bruker Daltonic Inc., Billerica, MA, USA) equipped with a pulsed flame photometric detector and a 394 nm sulfur filter was used to analyze TPTCL concentrations and their degradation products in samples. An HP-5 capillary column (30 m × 320 μm, 0.25 μm, Agilent Technologies Inc., Santa Clara, CA, USA) was used for separation. The injection volume was 1.0 μL and the injection port temperature was 250 °C. The chromatographic column temperature was set to 45 °C for 3 min, increased to 120 °C at a rate of 15 °C min^−1^ and held for 2 min, then ramped up to 150 °C at a rate of 5 °C min^−1^ and held for 2 min, further rose to 220 °C at a rate of 10 °C min^−1^ and held for 2 min, and eventually reached 240 °C at a rate of 10 °C min^−1^ and held for 3 min. The carrier gas was nitrogen (purity ≥ 99.999%) at a flow rate of 2.0 mL/min. The detection temperature was 280 °C. The make-up gas was hydrogen—(14 mL min^−1^) and air (27 mL min^−1^). The signal delay time was 4.0 ms and the pulse width was 20.0 ms.

For seawater samples, the recovery, relative standard deviation (RSD), and detection limit were 80.54–115.59%, 3.42–8.51%, and 194.1 ng L^−1^ for TPTCL, 72.94–87.30%, 4.40–13.20%, and 76.5 ng L^−1^ for DPTCL, and 79.57–118.48%, 1.23–10.98%, and 156 ng L^−1^ for MPTCL, respectively. For seaweed samples, the recovery, relative standard deviation (RSD), and detection limit were 102.89–107.98%, 6.58–8.24%, and 0.012 mg kg^−1^ for TPTCL, 73.12–76.14%, 0.68–8.47%, and 0.017 mg kg^−1^ for DPTCL, and 76.69–110.25%, 2.74–5.23%, and 0.010 mg kg^−1^ for MPTCL, respectively.

### 4.3. Statistical Analysis

The Kruskal–Wallis and Tukey’s HSD multiple comparison tests were used to analyze significant differences in the contents of TPTCL and their degradation products across different algal parts in each month and across cultivation periods, as not every dataset showed a normal distribution and homogeneous variance. Differences were considered significant at a probability of 5% (*p* < 0.05). All analyses were conducted using SPSS software (Version 26.0, IBM Corporation, Armonk, NY, USA).

## 5. Conclusions

In conclusion, the sporophyte of cultivated *Undaria pinnatifida* had an accumulation and degradation capacity for TPTCL. TPTCL contents and degradation products varied with the algal growth stages and algal parts. TPTCL accumulated in the blades at the growth stage and the blades, stipes, sporophylls, and holdfasts at the mature stage. The TPTCL content among algal parts was blades > holdfasts > sporophylls > stipes. The primary degradation product DPTCL accumulated only in the blades at any stage, and the secondary degradation product MPTCL accumulated in the blades at the growth stage and in the blades, stipe, and sporophyll at the mature stage. The MPTCL content among algal parts was blades > sporophylls > stipes. The accumulation pattern of TPTCL and its degradation products seems closely related to nutrient allocation in *U. pinnatifida*. Due to the limited data in this study, further studies are needed to identify TPTCL’s accumulation and degradation mechanisms in marine algae, especially with more attention paid to the functions of some key substances such as alginic acid.

## Figures and Tables

**Table 1:** Seasonal morphological characteristics of *U. pinnatifida* during field cultivation. Different lowercase letters represent significant differences in morphological characteristics among months.

Blade				Stipe			Sporophyll		
Month	Length (cm)	Width (cm)	Fresh Weight (g)	Length (cm)	Width (cm)	Fresh Weight (g)	Length (cm)	Width (cm)	Fresh Weight (g)
December	74.25 ± 11.27 ^a^	12.37 ± 4.20 ^a^	15.74 ± 6.84 ^a^	15.90 ± 4.44 ^a^	1.11 ± 0.30 ^a^	10.74 ± 3.46 ^a^	None	None	None
January	123.67 ± 16.94 ^c^	21.76 ± 6.39 ^bc^	48.79 ± 26.34 ^a^	30.62 ± 7.03 ^bc^	1.80 ± 0.37 ^bc^	42.41 ± 15.94 ^b^	None	None	None
February	138.33 ± 27.06 ^d^	22.54 ± 5.99 ^cd^	58.11 ± 22.73 ^a^	38.96 ± 10.78 ^c^	1.86 ± 0.29 ^cd^	63.48 ± 24.71 ^c^	6.83 ± 2.42 ^a^	3.45 ± 1.28 ^a^	2.45 ± 2.00 ^a^
March	89.64 ± 9.53 ^b^	15.72 ± 2.72 ^ab^	26.52 ± 7.50 ^a^	23.63 ± 7.83 ^ab^	1.49 ± 0.83 ^b^	26.30 ± 7.88 ^ab^	6.54 ± 3.03 ^a^	2.08 ± 0.34 ^a^	0.88 ± 0.41 ^a^
April	80.84 ± 12.99 ^ab^	28.60 ± 8.47 ^d^	67.33 ± 42.65 ^a^	22.47 ± 9.25 ^ab^	1.60 ± 0.31 ^bc^	36.71 ± 16.99 ^b^	11.59 ± 6.93 ^a^	3.34 ± 1.26 ^a^	6.99 ± 9.21 ^a^
May	101.88 ± 20.97 ^c^	36.89 ± 9.39 ^e^	247.36 ± 133.77 ^b^	36.61 ± 8.37 ^c^	2.21 ± 0.35 ^d^	93.73 ± 32.10 ^d^	28.02 ± 11.70 ^b^	6.49 ± 1.62 ^b^	105.95 ± 72.5 ^b^

## Data Availability

The data of this study are available from the corresponding author upon reasonable request.
